# Clinical, Dermoscopic and Reflectance Confocal Microscopy Characteristics Associated With the Presence of Negative Pigment Network Among Spitzoid Neoplasms

**DOI:** 10.1111/exd.70154

**Published:** 2025-08-27

**Authors:** Marco Spadafora, Francesca Farnetani, Stefania Borsari, Shaniko Kaleci, Dafi Porat, Silvana Ciardo, Ignazio Stanganelli, Caterina Longo, Giovanni Pellacani, Alon Scope

**Affiliations:** ^1^ Department of Surgery, Medicine, Dental Medicine and Morphological Sciences University of Modena and Reggio Emilia Modena Italy; ^2^ Azienda Unità Sanitaria Locale—IRCCS di Reggio Emilia Skin Cancer Center Reggio Emilia Italy; ^3^ Department of Dermatology University of Modena and Reggio Emilia Modena Italy; ^4^ The Kittner Skin Cancer Screening & Research Institute, Sheba Medical Center, Ramat Gan and School of Medicine Tel Aviv University Tel Aviv Israel; ^5^ Skin Cancer Unit IRCCS Istituto Romagnolo per Lo Studio Dei Tumori (IRST) ‘Dino Amadori’ Meldola Italy; ^6^ Dermatology Unit, Department of Clinical and Experimental Medicine University of Parma Parma Italy; ^7^ Dermatology Clinic, Department of Clinical Internal, Anesthesiological and Cardiovascular Sciences Sapienza University of Rome Rome Italy

**Keywords:** dermoscopy, histopathology, negative pigment network, reflectance confocal microscopy, spitz naevus, Spitzoid melanoma

## Abstract

Negative pigment network (NPN) is a dermoscopic structure frequently associated with melanoma. Though commonly observed in Spitz naevi (SN) and Spitzoid melanoma (SM), its reflectance confocal microscopy (RCM) correlates have been primarily studied in non‐Spitzoid melanocytic neoplasms. This study aimed to identify clinical, dermoscopic, and RCM features associated with dermoscopic NPN in Spitzoid neoplasms and explore its histopathological correlates. We retrospectively analysed clinical, dermoscopic, and RCM images from 128 histopathologically confirmed SN and SM cases diagnosed between 2014 and 2020. Lesions were grouped by presence or absence of dermoscopic NPN, and comparisons were made across clinical, dermoscopic, and RCM features. A subset of 20 cases underwent histopathologic correlation. Of the 128 cases, 96 (74%) were SN and 32 (26%) SM. NPN was present in 58 lesions (45%)—40 SN (42%) and 18 SM (56%). NPN was associated with lesion diameter ≥ 5 mm, presence of shiny white structures, dotted vessels, and inversely associated with diffuse blue‐white veil. SMs showed higher frequencies of asymmetry, multicomponent patterns, and extensive NPN. RCM features previously linked to NPN—round or linear surface disruptions, bright suprabasal areas, and broadened interpapillary spaces—were seen in 87% of cases but did not correlate with diagnosis or dermoscopic NPN. Corresponding histologic features included keratin‐filled dells, hypergranulosis, and broadened rete ridges or infundibula. RCM correlates of dermoscopic NPN are frequently observed in Spitzoid neoplasms, independent of visible dermoscopic NPN, suggesting perceptibility may depend on contrast within dermoscopic patterns.

## Graphical abstract

Correlates of negative pigment network in Spitzoid tumors.

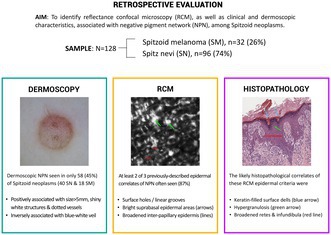

## 1 Introduction

Melanoma diagnosis has been improved by recognition of specific dermoscopic structures [1]. Negative pigment network (NPN) is a dermoscopic structure defined as ‘serpiginous, interconnecting, hypopigmented lines that surround irregularly shaped brown structures, which resemble elongated and curvilinear globular structures’ [2, 3]. The presence of NPN has high specificity (95%), albeit low sensitivity (22%), for melanoma diagnosis [4, 5].

The microscopic tissue substrates of NPN had been, to some extent, elusive [6, 7]. Recently, we reported on the reflectance confocal microscopy (RCM) findings underlying NPN, in a series of 21 melanomas and 29 naevi, of which two were Spitz naevi (SN) [8]. Dermoscopy to RCM correlation and comparison to histopathological findings showed that NPN is associated with an abnormal proliferation of both atypical melanocytes and surrounding keratinocytes. The hypopigmented lines of NPN correlated with broadened epidermal rete ridges and infundibula, which often display overlying surface dells and wedge‐shaped hypergranulosis; the pigmented curvilinear globular structures of NPN correlated with a proliferation of melanocytes along and between thin elongated epidermal retes [8].

The RCM and histopathological tissue alterations underlying NPN were reminiscent of those commonly seen among Spitzoid neoplasms. Notably, NPN has been frequently observed among SN and among melanomas with Spitzoid features (‘Spitzoid melanoma’, SM) [3, 4, 9, 10, 11, 12, 13, 14, 15]. To that end, we decided to focus our research of NPN on Spitzoid neoplasms. The primary aim of the present study was to identify clinical, dermoscopic, and RCM characteristics associated with the presence of NPN in SN and SM. In an exploratory fashion, we also compared the RCM and histopathological findings in a subset of the lesions.

## 2 Methods

The study was approved by the Institutional Ethics Committee AVEN, in accordance with the ethical standards on human experimentation and with the Declaration of Helsinki.

### 2.1 Study Dataset

The study was a retrospective analysis. We searched the databases of three Italian tertiary skin cancer centers for cases archived between the years 2014 and 2020. Included cases were those with (1) histopathologically‐confirmed diagnosis of SN or SM, and (2) available images acquired using standardised clinical, dermoscopic, and RCM imaging; the RCM image set had to include at least three mosaic images acquired at suprabasal epidermal level, basal epidermal/superficial dermal‐epidermal junction (DEJ), and deeper DEJ/superficial dermis. Lesions were excluded if they had an equivocal histopathological diagnosis, such as ‘atypical Spitzoid lesion’, [16] or if quality of their images was poor and precluded accurate image analysis.

### 2.2 Image Acquisition and Analysis

Clinical images were taken with a Canon G16 camera (Cannon, Tokyo, Japan). Standardised polarised dermoscopic images were obtained with DermLite Photo (DermLite LLC, Aliso Viejo, CA, USA). RCM images were acquired with a VivaScope1500 (Mavig GmbH, Munich, Germany).

Image analysis was performed using clinical, dermoscopic, and RCM parameters (Table S1) [3, 4, 17, 18, 19, 20]. Lesions from the study dataset were evaluated in a random order. The first 20 cases, including SN (*n* = 17) and SMs (*n* = 3), were jointly evaluated by two readers (MS and AS) to refine the study criteria and to train the main reader. The subsequent set of 108 cases, which included SN (*n* = 79) and SMs (*n* = 29), was evaluated by a single reader (MS), blinded to the final histopathological diagnosis. Any uncertain or ambiguous findings encountered by the main reader (MS) during image evaluation were subsequently discussed and resolved by consensus with the senior reader (AS).

### 2.3 Histopathological Subset Analysis

The exploratory RCM‐histopathological comparison was performed on a subset of the study lesions, including SN (*n* = 13) and SMs (*n* = 7). Histopathological slides were digitally scanned and viewed side‐by‐side and at a comparable level of magnification along with the corresponding RCM images. This subset was selected based on the availability of high‐quality RCM mosaics and corresponding well‐oriented histopathological slides that allowed for reliable anatomic‐level correlation. For each RCM feature, we identified the precise anatomic level of imaging and searched for the most likely corresponding histopathological features at that anatomic level.

### 2.4 Statistical Analysis

Statistical analysis was performed using STATA software version 17 (StataCorp LP, College Station, TX, USA). Descriptive statistics were presented for baseline demographic clinical characteristics for the entire dataset, as well as for subgroups with and without dermoscopic NPN. Continuous variables were presented as the number of patients (*N*), mean, standard deviation (SD), minimum (min), and maximum (max) and compared between subgroups using unpaired‐Student's‐t test; categorical variables were presented as frequency (*N*, percentage [%]) and compared using Pearson's‐chi‐squared test. A multivariate logistic regression model was carried out using a stepwise selection method to compare predictive factors between cases and controls. In the first step, the intercept‐only model was fitted and individual score statistics for the potential variables were evaluated. A significance level of *p* < 0.05 was used to allow a variable into the model. In stepwise selection, an attempt was made to remove any insignificant variables from the model, before adding a significant variable to the model. Hosmer and Lemeshow tests were used to evaluate the ‘goodness of fit’ in the selection model. Data from the univariate and multivariate logistic regression analyses were expressed as odds ratio (OR) and 95% confidence interval (CI). A *p* < 0.05 was considered statistically significant.

## 3 Results

### 3.1 Clinical, Dermoscopic and RCM Findings and Comparison Between SN and SM2

The pertinent demographic, clinical and dermoscopic data are shown (Table S2). The study dataset included 96 SN (74%) and 32 SMs (26%), contributed by 128 patients. The mean age was 35 years (range 3–81 years); there was female preponderance (67%), and lesions were most frequently located on the lower extremities (54%). The descriptive data of RCM findings by diagnosis is also shown (Table S3).

We first sought to identify clinical, dermoscopic, and RCM discriminatory parameters between SN and SMs. On univariate analysis (Table S4), clinical parameters that were associated with the diagnosis of SM included tan to light brown colour (referent category ‘skin colour’, OR 2.65, *p* = 0.04) and diameter ≥ 10 mm (referent category ‘< 5 mm’, OR 4.12, *p* = 0.02). Dermoscopic parameters predictive of SM diagnosis included presence of dermoscopic NPN (OR 2.3, *p* = 0.04) and pattern asymmetry (OR 3.66, *p* = 0.02), while a pigmented‐homogenous pattern was associated with SN diagnosis (referent category ‘multicomponent pattern’, OR 0.25, *p* = 0.05). None of the RCM parameters was associated with diagnosis. In addition, the multivariate model showed an overall poor discrimination between SN and SMs (Table S4).

### 3.2 Clinical and Dermoscopic Parameters Associated With Presence of Dermoscopic NPN

**FIGURE 1:**
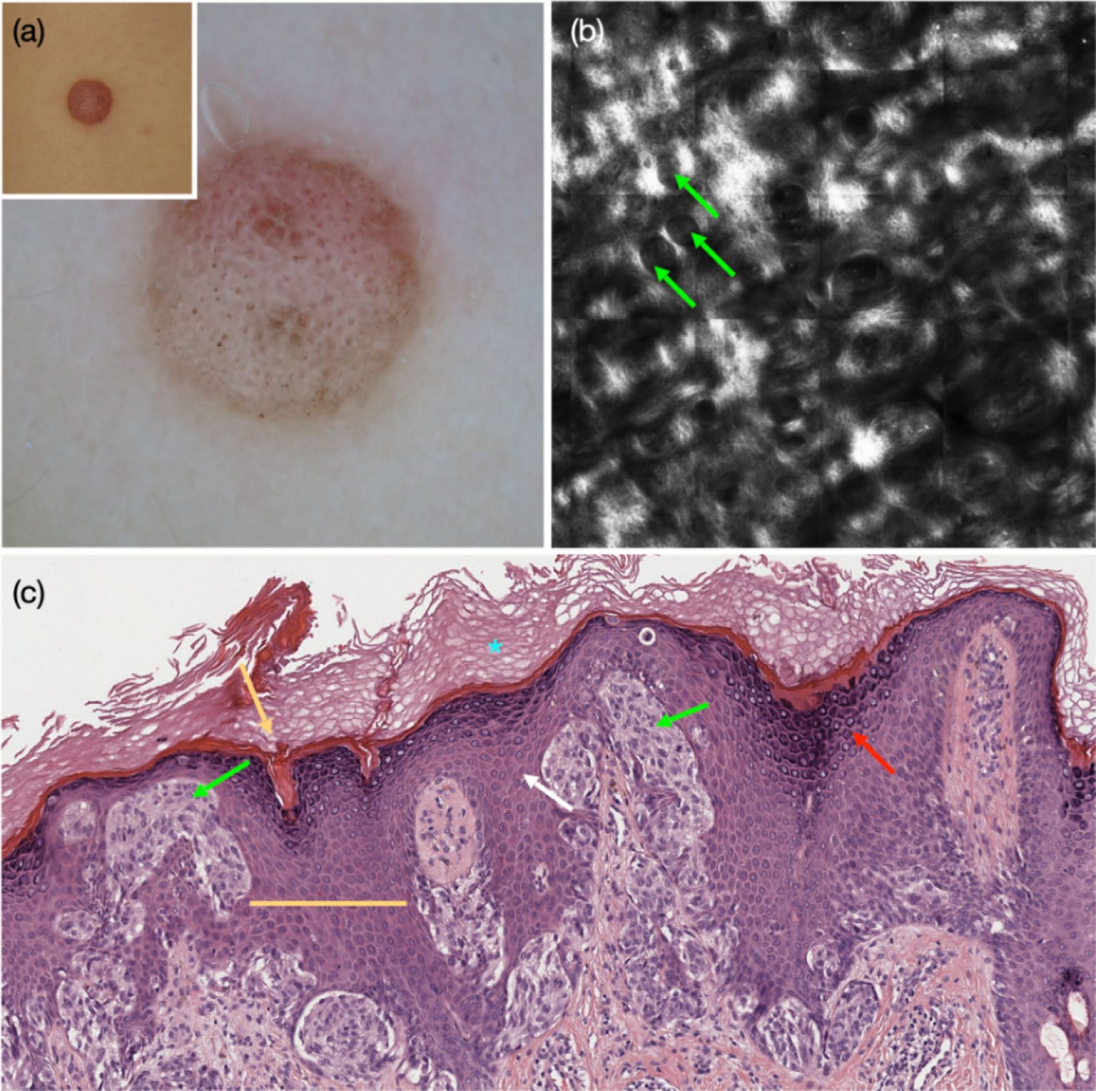
Spitz naevus, on the forearm of a 10‐year‐old patient boy. (a) Inset showing a light brown nodule. Dermoscopy showing globular pattern with diffuse negative pigment network, shiny white structures, and dotted vessels. (b) RCM at the spinous‐granular layers shows bright suprabasal epidermal areas, and junctional nests (green arrows) protruding into the suprabasal epidermis that appear darker than the surrounding epidermis. (c) Corresponding histopathology shows hyperkeratosis (blue asterisk), keratin‐filled surface dells (yellow arrow), wedge‐shaped and diffuse hypergranulosis (red arrow), acanthosis (white arrow) and broadened retes (yellow line). Some of the junctional nests protrude into‐ and appear immersed within‐ the spinous layer of the epidermis (green arrows).

**FIGURE 2:**
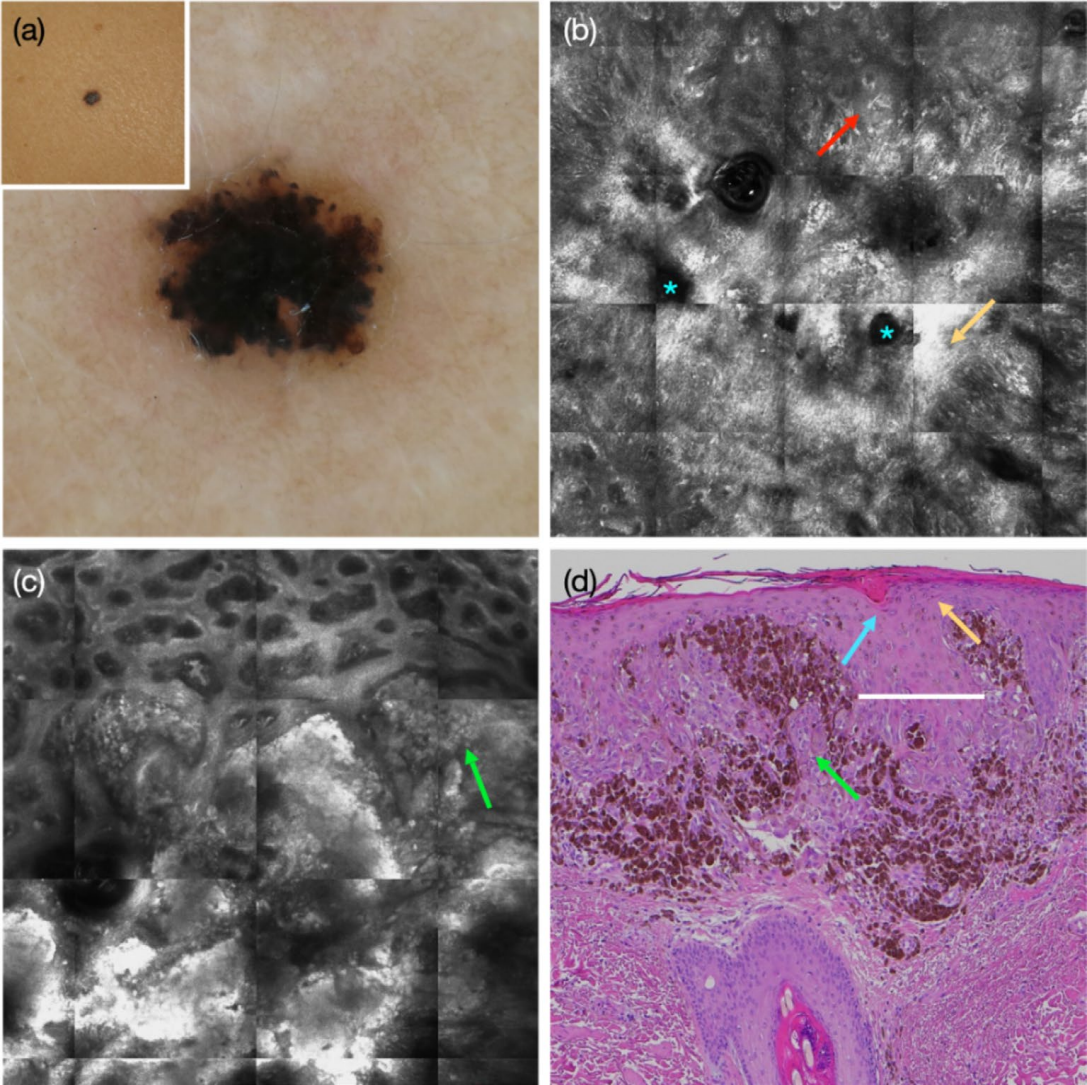
Spitzoid melanoma 0.6 mm in thickness, on the upper trunk of a 31‐year‐old woman. (a) Inset showing a small black papule. Dermoscopy showing diffuse dark blue‐black homogenous pattern with few globules and pseudopods at the periphery, while a negative pigment network is not seen. (b) RCM at the spinous‐granular layers shows round surface holes (blue asterisks), bright suprabasal epidermal areas (yellow arrow), and multiple dendritic cells in a pagetoid pattern (red arrow). (c) RCM at the DEJ level shows clod pattern composed of large confluent nests, with a non‐homogeneous brightness, filling and expanding the dermal papillae (green arrow). (d) Corresponding histopathology shows keratin‐filled surface dells (blue arrow), focal hypergranulosis (yellow arrow), and confluent aggregates of pigmented and non‐pigmented epithelioid melanocytes (green arrow), separated by acanthotic epidermis with broadened retes (white line).

**FIGURE 3:**
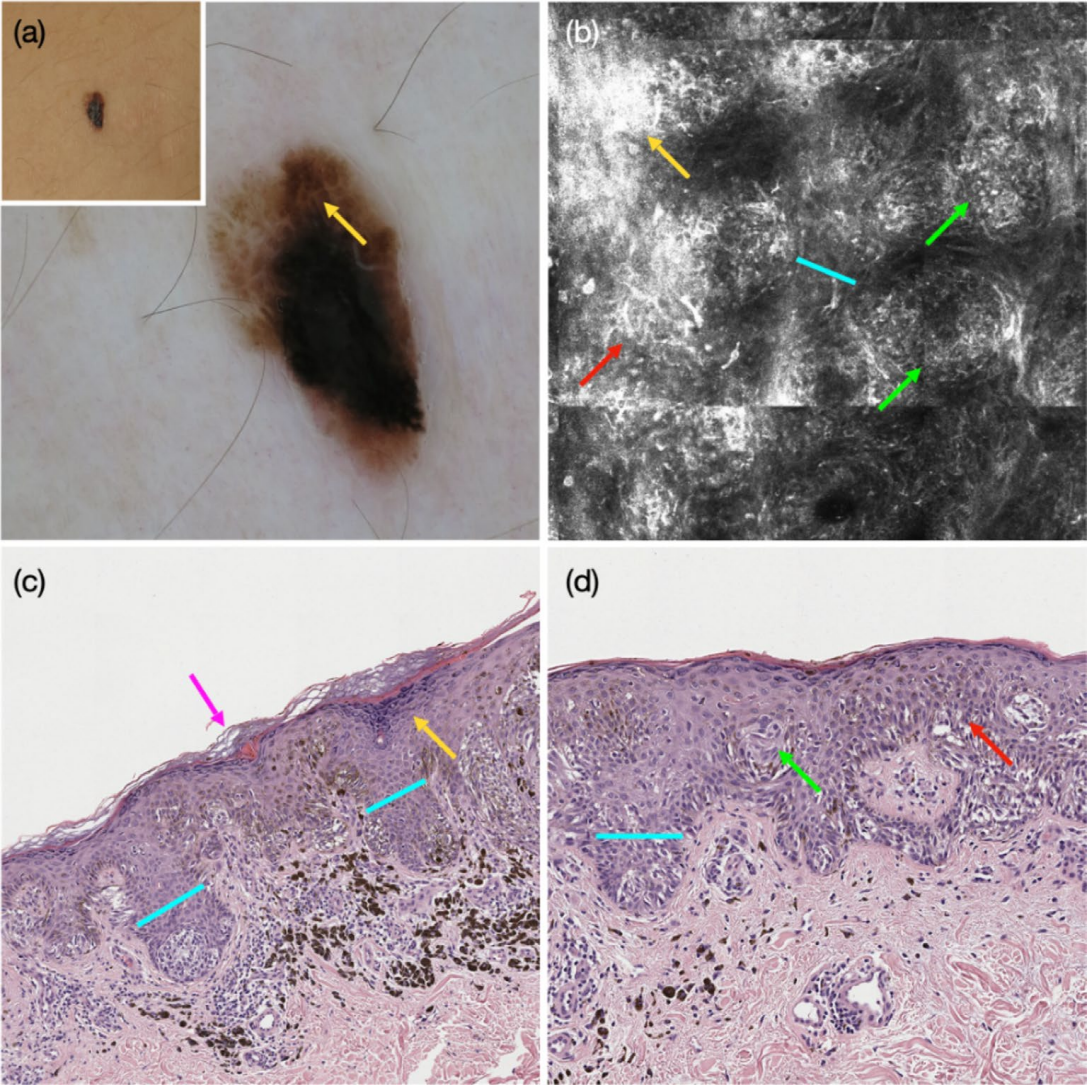
Spitzoid melanoma 0.6 mm in thickness, on the abdomen of a 41‐year‐old woman. (a) Inset showing an asymmetric black and brown papule. Dermoscopy shows an asymmetric multicomponent pattern with foci displaying a negative pigment network (yellow arrow). (b) RCM at the spinous‐granular layers showing bright suprabasal epidermal areas (yellow arrow), broadened interpapillary epidermis (blue line), and multiple dendritic cells (red arrow). There are multiple bright dishomogeneous junctional nests that protrude into‐ and appear ‘immersed’ within‐ the epidermis (green arrows). Corresponding histopathology showing (c, d) keratin‐filled surface dells (purple arrow), wedge‐shaped hypergranulosis (yellow arrow), broadened retes (blue lines), melanocytes in pagetoid pattern (red arrow), and junctional nests that are appeared immersed within the surrounding spinous keratinocytes (green arrow).

Dermoscopic NPN was seen in 58 of the 128 lesions (45%), including 40 SN (42%) and 18 SMs (56%). Clinical parameters associated with the presence of NPN included the diagnosis of SM compared with SN (OR = 2.33, *p* = 0.04) and lesion diameter ≥ 5 mm (compared to referent category ‘< 5 mm’: for diameter ≥ 5 mm and < 10 mm, OR = 4.84, *p* = 0.01; and for ≥ 10 mm OR = 27.4, *p* < 0.001) and nodular compared with flat contour (OR = 6.24, *p* = 0.008) (Table 1). Dermoscopic parameters associated with the presence of NPN included dermoscopic asymmetry (OR = 2.32, *p* = 0.04); lightly pigmented colour (OR = 4.97, *p* = 0.002, Figure 1), and amelanotic colour (OR = 4.39, *p* = 0.02) compared to pigmented colour (Figure 2); amelanotic homogeneous pattern (OR = 14.25, *p* = 0.01) and multi‐component pattern (OR = 11.4, *p* = 0.01, Figure 3), compared to globular pattern; and the presence of shiny white structures (SWS) (OR = 9.62, *p* < 0.001, Figure 1, Figures S2 and S3) and of dotted vessels (OR = 59.7, *p* < 0.001, Figure 1, Figures S2 and S3) (Table 1). We observed that SWS mostly coincided with, and highlighted, the hypopigmented areas of the NPN (Figure 1, Figure S2), while dotted vessels were mostly located within the globular portion of the NPN (Figure 1, Figure S3). In contrast, the diffuse presence of blue‐white veil (≥ 30% of the lesional area) was negatively associated with the presence of NPN (OR = 0.30, *p* = 0.008) (Figure 2).

**TABLE 1:** Univariate and multivariate models for presence vs. absence of dermoscopic NPN by clinical and dermoscopic parameters.

		Univariate analysis		Multivariate analysis	
		OR 95% CI	*p*		
*Clinical parameters*					
Diagnosis	Spitz naevus	Ref.			
	Spitzoid melanoma	2.33 (1.02–5.31)	**0.044**		
Size	< 5 mm	Ref.		Ref.	
	≥ 5 mm, < 10 mm	4.84 (1.88–12.48)	**0.001**	10.03 (1.89–53.03)	**0.007**
	≥ 10 mm	27.4 (6.30–119.36)	**< 0.001**	39.21 (4.37–351‐88)	**0.001**
Location	Head and neck	Ref.			
	Trunk	1.46 (0.22–9.48)	0.688		
	Upper extremity	2.15 (0.33–13.80)	0.418		
	Lower extremity	1.83 (0.31–10.67)	0.500		
Colour	Skin colour	Ref.			
	Pink to red	4.66 (0.22–97.49)	0.321		
	Light brown to tan	2.44 (0.13–43.47)	0.543		
	Medium brown to black	0.41 (0.02–6.97)	0.544		
Palpability	Flat	Ref.			
	Palpable	1.38 (0.64–2.93)	0.402		
	Nodular	6.24 (1.58–24.34)	**0.008**		
*Dermoscopic parameters*					
Colour	Darkly pigmented	Ref.			
	Lightly pigmented	4.97 (1.70–13.86)	**0.002**		
	Amelanotic	4.39 (1.27–15.11)	**0.019**		
Blue‐white structures	Absent	Ref.		Ref.	
	< 30%	0.56 (0.23–1.32)	0.190	0.23 (0.05–0.98)	**0.047**
	≥ 30%	0.30 (0.12–0.73)	**0.008**	0.21 (0.05–0.96)	**0.044**
Shiny white structures	Absent	Ref.		Ref.	
	Present	9.62 (4.17–22.19)	**< 0.001**	9.11 (2.62–31‐66)	**0.001**
Pattern type	Globular	Ref.			
	Starburst	—			
	Reticular	2.5 (0.61–10.22)	0.202		
	Pigmented homogeneous	0.88 (0.22–3.53)	0.876		
	Amelanotic homogenous	14.25 (2.98–68.01)	**0.001**		
	Multicomponent	11.4 (2.5–51.10)	**0.001**		
Pattern symmetry	Symmetric	Ref.			
	Asymmetric	2.32 (1.04–5.18)	**0.039**		
Blood vessels	Absent	Ref.		Ref.	
	Dotted	59.70 (7.70–462.74)	**< 0.001**	53.12 (4.15–681.20)	**0.002**
	Linear	—		—	—
	Polymorphous	1.72 (0.36–8.22)	0.496	0.144 (0.01–1.45)	0.101
	Other	9.18 (0.98–86‐05)	0.052	12.88 (0.44–370.15)	0.136

*Note:* Bold values indicate statistical significant parameters.

Abbreviations: CI, confidence interval; OR, odds ratio.

The multivariate model for clinical and dermoscopic parameters showed that a diameter ≥ 5 mm and the presence of SWS and dotted vessels were positively associated with NPN, while the presence of blue‐white veil was negatively associated with NPN (Table 1).

### 3.3 Association Between RCM Parameters and Dermoscopic NPN

Several RCM epidermal criteria were highly prevalent among the dataset of 128 Spitzoid neoplasms (Table S3). These included round surface holes and/or elongated surface grooves (*n* = 96, 75%, Figure 2, Figure S1), bright suprabasal epidermal areas (*n* = 74, 58%, Figures 2 and 3, Figures S1–S3), and broadened inter‐papillary epidermis (*n* = 99, 77%, Figure 3). Only 6 of 128 Spitzoid neoplasms (4%) lacked all three of these epidermal criteria, while 11 (9%) showed one, 64 (50%) two, and 47 (37%) showed all three of these RCM epidermal criteria. There was no significant difference in the frequency of these parameters between SN and SM (Table S3).

We analysed RCM parameters that were associated with the presence of dermoscopic NPN. These included linear surface grooves (OR = 2.30, *p* = 0.02), round surface holes (OR = 2.91, *p* = 0.016), and bright suprabasal epidermal areas (OR = 2.94, *p* = 0.004). An atypical epidermal pattern was negatively associated with the presence of dermoscopic NPN (OR = 0.19, *p* = 0.004) (Table 2). However, the multivariate model showed that these RCM epidermal parameters were non‐significant predictors for the presence vs. absence of dermoscopic NPN (Table 2).

**TABLE 2:** Univariate and multivariate models for presence versus absence of dermoscopic NPN by RCM parameters.

RCM parameters		Univariate analysis		Multivariate analysis	
		OR 95% CI	*p*	OR 95% CI	*p*
Linear surface grooves	Absent	Ref.			
	Present	2.30 (1.11–4.74)	**0.024**		
Round surface holes	Absent	Ref.			
	Present	2.91 (1.21–6.69)	**0.016**		
Predominant epidermis pattern	Typical	Ref.		Ref.	
	Atypical	0.19 (0.08–0.48)	**< 0.001**	0.18 (0.04–0.77)	0.21
	Disarranged	0.30 (0.08–1.16)	0.083	1.08 (0.16–7.15)	0.93
Bright suprabasal epidermal areas	Absent	Ref.			
	Present	2.94 (1.40–6.14)	**0.004**		
Roundish cells in suprabasal layer	Absent	Ref.			
	< 30%	0.80 (0.29–2.15)	0.661		
	≥ 30%	2.20 (0.19–25.07)	0.524		
Dendritic cells in suprabasal layer	Absent	Ref.			
	< 30%	1.00 (0.45–2.18)	1.000		
	≥ 30%	0.90 (0.32–2.47)	0.838		
Predominant DEJ pattern	Thin ring	Ref.			
	Thick ring	2.67 (0.67–10.55)	0.160		
	Meshwork	1.42 (0.434–5.94)	0.629		
	Clods	1.31 (0.31–5.53)	0.711		
	Nonspecific/disarranged	0.17 (0.01–1.91)	0.154		
Interpapillary epidermis	Regular	Ref.			
	Broadened	1.47 (0.52–4.12)	0.455		
Atypical cells at DEJ	Absent	Ref.			
	< 30%	0.85 (0.36–2.00)	0.713		
	≥ 30%	1.11 (0.37–3.28)	0.844		
Nests—predominant type	Dense homogeneous	Ref.		Ref.	
	Dense and sparse	1.16 (0.31–4.28)	0.823	1.2 (0.08–17.8)	0.87
	Heterogeneous/atypical	2.23 (0.91–5.47)	0.079	23.1 (2.3–228.4)	**0.007**
Nests—predominant location	Junctional	Ref.			
	Dermal	3.33 (0.81–13.59)	0.093		
	Both	1.25 (0.36–4.24)	0.721		
DP‐nests predominant pattern	Dark	Ref.		Ref.	
	Bulging	0.65 (0.27–1.52)	0.323	10.5 (1.1–95.7)	**0.037**
	Expanded	3.00 (0.72–12.35)	0.128	374.0 (6.1–22793.6)	**0.005**
	Nonspecific	0.40 (0.11–1.43)	0.161	2.84 (0.01–540.1)	0.696
Dark	< 30	Ref.		Ref.	
	≥ 30	1.51 (0.67–3.37)	0.316	920.4 (24.8–34029.0)	**< 0.001**
Bulging	< 30	Ref.			
	≥ 30	0.64 (0.30–1.37)	0.258		
Expanded	< 30	Ref.			
	≥ 30	1.63 (0.62–4.26)	0.316		
Nonspecific	< 30	Ref.			
	≥ 30	1.07 (0.49–2.32)	0.862		
DP‐ring pattern	Edged	Ref.			
	Non‐edged	0.66 (0.28–1.55)	0.342		
	Mixed	0.62 (0.20–1.88)	0.404		

*Note:* Bold values indicate statistical significant parameters.

Abbreviations: CI, confidence interval; DEJ, dermal‐epidermal junction; DP, dermal papilla; OR, odds ratio; RCM, reflectance confocal microscopy.

### 3.4 Comparison of RCM Criteria and Histopathological Findings

We compared the presence of RCM epidermal parameters with the histopathological findings in a subset of 20 lesions, including SN (*n* = 13) and SMs (*n* = 7). We observed that (1) round surface holes and/or elongated surface grooves on RCM most likely corresponded with keratin‐filled surface invaginations/dells on histopathology (Figure 2, Figure S1); (2) bright suprabasal epidermal areas on RCM most likely correspond with wedge‐shaped or diffuse hypergranulosis (Figures 1 and 3, Figure S2); and (3) broadened inter‐papillary epidermis correspond with broadened epidermal retes and infundibula (Figure 3, Figure S2). We found that histopathological criteria indicating epidermal hyperplasia were highly prevalent among Spitzoid neoplasms, including surface orthokeratosis (75%), surface dells (80%), hypergranulosis (55%), epidermal acanthosis (85%) and presence of broadened epidermal retes and infundibula (85%) (Table S5). There was no significant difference in the frequency of these parameters between SN and SMs.

Finally, we explored the RCM attributes related to the proliferation of melanocytes and compared them with the histopathological findings. Under RCM, the junctional melanocytic nests appeared to protrude into the suprabasal epidermis, were not well demarcated from and were closely apposed to the surrounding spinous‐granular keratinocytes (Figures 1, 2, 3, Figure S3). In some cases, the nests appeared less reflective than the surrounding keratinocytes (Figure 2, Figure S3). These findings corresponded on histopathology to vertically oriented junctional nests that appeared within the stratum spinosum (Figures 1, 2, 3, Figure S3).

## 4 Discussion

The epidermal RCM criteria, which were previously shown to underlie dermoscopic NPN in the context of non‐Spitzoid naevi and melanoma, [8] are commonly and diffusely seen among Spitzoid neoplasms. We found that 96% of Spitzoid neoplasms displayed at least one of the RCM epidermal criteria, including round surface holes and/or elongated surface grooves, bright suprabasal epidermal areas, and broadened inter‐papillary epidermis. A third of cases showed all three RCM epidermal criteria. The histopathological findings, which appeared to best correspond to these RCM criteria, were also highly prevalent and included keratin‐filled surface dells, wedge‐shaped or diffuse hypergranulosis, and broadened infundibula and rete ridges.

The aforementioned epidermal changes, seen under RCM, as well as histopathology, should have translated to the presence of NPN under dermoscopy. However, dermoscopic NPN was discernible in only 45% of study lesions, including 42% of SN and 56% of SM. To better understand this RCM–dermoscopic discrepancy, we looked for clinical and dermoscopic predictors for the presence of dermoscopic NPN. We found that NPN is more apparent in lesions ≥ 5 mm in diameter, and with the dermoscopic presence of SWS and of dotted vessels, while the presence of blue‐white veil was negatively associated with perceptibility of NPN. Hence, we conjecture that among Spitzoid neoplasms, the visibility of the NPN depends on clinical and dermoscopic colours and structures, which enhance or obscure contrast between the hypopigmented lines and the globular structures of NPN. For example, the diffuse pigmentation in blue‐white veil likely obscures the NPN. In contrast, in lightly‐ to non‐pigmented Spitzoid neoplasms, stromal parameters, including the presence of SWS and dotted vessels, may be more perceptible under dermoscopy and facilitate the delineation of NPN. The SWS tend to coincide with the hypopigmented lines of NPN, whereas dotted vessels coincide and accentuate the globular structures of NPN [7]. Hence, our study demonstrates an important limitation of dermoscopy in the bedside recognition of the tissue changes that underlie NPN in a subset of melanoma. This observation may account for the relatively low sensitivity of NPN in melanoma diagnosis.

While the epidermal criteria mostly contribute to the hypopigmented lines of dermoscopic NPN, the melanocytic proliferation is aligned with the globular structures of NPN. We observed that the nested melanocytic proliferation was closely apposed to the surrounding epidermal keratinocytes. Junctional nests often protruded into and appeared immersed within the suprabasal epidermis. Previous studies reported epidermal hyperplasia to be a common finding among SN [21]. Two retrospective studies of SN, one enrolling 349 cases from Spain and Germany and another including 130 cases from Puerto Rico, observed epidermal hyperplasia in about two thirds of cases, mostly junctional and compound SN [22, 23]. The expression of a proliferation marker, proliferating cell nuclear antigen (PCNA), was shown to be increased in both melanocytes and basal keratinocytes of Spitz naevi, but not in non‐lesional keratinocytes nor in keratinocytes of non‐Spitz naevi [24]. Taken together, we postulate that the neoplastic melanocytes in Spitzoid neoplasms induce the closely accompanying keratinocytic hyperplasia, possibly to facilitate tumour growth.

**FIGURE 4:**
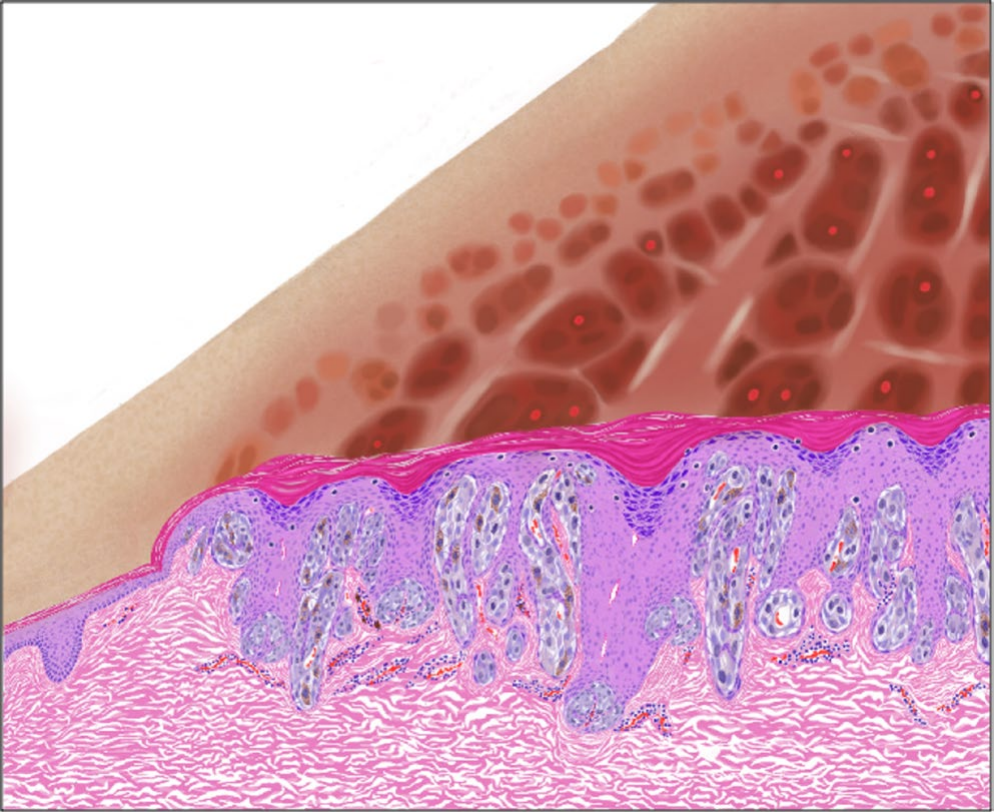
Model of dermoscopy to histopathology correlation of negative pigment network (NPN) in Spitzoid neoplasms. Under dermoscopy, the NPN is composed of interconnecting hypopigmented lines, which surround round to elongated, pigmented globular structures. The hypopigmented lines may contain shiny white structures, while the globular structures may be composed of pigmented dots and dotted vessels. Under histopathology, the hypopigmented lines of the dermoscopic NPN are aligned with keratin‐filled surface dells, wedge‐shaped hypergranulosis and broadened retes and infundibulae. The surrounding dermal fibroplasia likely correlates with the dermoscopic shiny white structures. The globular structures of the dermoscopic NPN are aligned with melanocytic nests, between epidermal cords. Notably, the nests may protrude high into the overlying epidermis. The nests are closely cuffed by the surrounding keratinocytes and are accompanied by dilated blood vessels. We conjecture that, while these histopathological findings are common among Spitzoid neoplasms, their dermoscopic manifestation as NPN depends on contrast between the tissue elements that contribute to the hypopigmented vs. the globular portions of the NPN.

Integrating the clinical, dermoscopic, RCM, and histopathological findings, we propose a model for the dermoscopy to histopathology correlation of NPN in Spitzoid neoplasms. The model demonstrates the alignment of the aforementioned epidermal criteria with the hypopigmented lines of NPN, while the melanocytic proliferation that is interwoven with thin elongated epidermal retes is aligned with the globular portion of NPN (Figure 4).

Finally, in line with previous publications [19, 25, 26, 27, 28] our study reiterates the difficulty in differentiation between SM and SN. Some clues suggestive of SM diagnosis emerged on univariate analysis, including clinical parameters of larger size and lightly pigmented colour, and dermoscopic parameters of asymmetry, multicomponent pattern, and presence of NPN. However, the multivariate model showed poor differentiation between SM and SN, supporting the prevailing clinical practice of excising Spitzoid neoplasms in adults [17].

Our study has limitations. First, this was a moderately sized dataset, as Spitzoid neoplasms, and particularly SM, are infrequently encountered in clinical practice. Second, we did not include atypical spitzoid lesions, and therefore, our findings are not helpful in characterising this subgroup of grey zone spitzoid lesions.

Second, while the dataset originated from three medical centers, all are from Italy, potentially limiting generalisability to other populations, also because of different skin phototype. Third, the RCM‐histopathological correlation was based on a limited subset of cases and on comparison of RCM and histopathological findings for the most likely fit, rather than a precise spatial correlation of microscopic findings. Finally, RCM evaluation is inherently limited in depth of penetration, and evaluation of dermal criteria was more restricted by the acanthotic epidermis of Spitzoid neoplasms.

In conclusion, the previously described RCM epidermal correlates of NPN are more prevalent among Spitzoid neoplasms than predicted by the dermoscopic presence of NPN. Our findings expand the definition of NPN by highlighting that its dermoscopic visibility depends not only on the presence of characteristic histopathologic and RCM features but also on the contrast‐enhancing role of specific clinical and dermoscopic parameters. Perceptibility of NPN under dermoscopy may be affected by lesion size and contrast between dermoscopic structures and colours. This dermoscopic limitation may account for the low sensitivity of NPN in melanoma diagnosis. Our findings require validation in Spitzoid neoplasms originating from different populations.

## Author Contributions

Conceptualization: Giovanni Pellacani and Alon Scope; data curation: Marco Spadafora, Silvana Ciardo, and Dafi Porat; formal analysis: Marco Spadafora and Shaniko Kaleci; supervision: Francesca Farnetani, Caterina Longo, and Alon Scope; investigation: Marco Spadafora and Alon Scope; writing – original draft: Marco Spadafora and Alon Scope; writing – review and editing: Caterina Longo, Alon Scope, Ignazio Stanganelli, Stefania Borsari, and Giovanni Pellacani.

## Ethics Statement

The study was approved by the local ethics committee in accordance with ethical standards on human experimentation and with the Declaration of Helsinki. The patients in this manuscript have given written informed consent to the publication of their case details.

## Consent

The authors consent to the publication of this submission (manuscript and figures).

## Conflicts of Interest

The authors declare no conflicts of interest.

## Supporting information


**Appendix S1:** Supporting Information.

## Data Availability

The data that support the findings of this study are available on request from the corresponding author. The data are not publicly available due to privacy or ethical restrictions.
