# Supplementary material for: Clinical, Dermoscopic and Reflectance Confocal Microscopy Characteristics Associated With the Presence of Negative Pigment Network Among Spitzoid Neoplasms
Source: Exp Dermatol. 2025 Aug 27;34(8):e70154. doi: 10.1111/exd.70154 (PMC12381913; doi:10.1111/exd.70154)
Supplement: Supplementary file 1 — Appendix S1: Supporting Information. [file EXD-34-e70154-s001.docx]

**SUPPLEMENTARY MATERIALS**

**eTable 1. Definition of dermoscopic and RCM parameters**

| DERMOSCOPIC PARAMETER | | DEFINITION |  |
| --- | --- | --- | --- |
| Colour | Darkly pigmented: lesion shows brown to black pigmented structures (globules, pigmented network, blotches, streaks) | |  |
|  | Lightly pigmented: lesion shows tan to light brown pigmented structures (globules, pigmented network, blotches, streaks) | | |
|  | Amelanotic: lesion shows pink to skin-coloured homogeneous pattern with none to minimal presence of pigmented structures | | |
| Blue-White Veil | An irregularly-shaped bluish blotch with an overlying whitish ground-glass haze | |  |
| Negative Pigment Network | Serpiginous interconnecting broadened hypopigmented lines ,which surround round, elongated and curvilinear globules | |  |
| Shiny White Structures | Discrete white lines or strands oriented parallel and/or orthogonal (perpendicular) to each other, seen only under polarized dermoscopy | |  |
| Dermoscopic pattern | Globular: numerous, variously-sized, round to oval structures with various shades of brown to grey-black | |  |
|  | Starburst: peripheral globules, pseudopods, or linear streaks, or a combination of them, located around most to all of the perimeter of the lesion | | |
|  | Reticular: grid-like pattern consisting of intersecting pigmented lines and hypopigmented holes | | |
|  | Pigmented-homogeneous: diffuse, brown, grey-blue to grey-black pigmentation in the absence of other distinctive local structures | | |
|  | Amelanotic-homogeneous: pink to skin-coloured structureless background, often associated with diffusely distributed vessels (dotted/linear/coiled/hairpin) | | |
|  | Multicomponent: A pattern composed of more than one of the above-described patterns | | |
| Blood Vessels | Dotted: pinpoint vessels | |  |
|  | Linear: elongated straight to mildly curved ("snake-like") vessels | | |
|  | Other: vessels that differ from the previously mentioned (e.g., coiled or hairpin) | | |
|  | Polymorphous: multiple types of vessels are present (e.g., dotted and linear) | | |
| RCM PARAMETER |  | |  |
| Linear surface grooves | Elongated dark furrows at the surface of the epidermis | |  |
| Round surface holes | Circular to oval dark areas or openings at the surface of the epidermis | |  |
| Epidermal patterns | Typical honeycomb/cobblestone pattern: an organized grid pattern formed by polygonal cells with thin bright outlines and dark nuclei or organized pattern of aggregated bright, small, polygonal cells separated by a less refractive border | |  |
|  | Atypical honeycomb/cobblestone pattern: a disorganized grid pattern formed by polygonal cells with variable size and shape of the bright cell outline and the dark nuclei or disorganized pattern of aggregated bright cells separated by a less refractive border, with variability in the size and shape of the cells | | |
|  | Disarranged: An epidermis lacking a recognizable honeycombed or cobblestone patterns, often with unevenly distributed bright granular particles and cells. | | |
| Pagetoid infiltration | Presence of bright round and/or dendritic-nucleated cells at the suprabasal (spinous-granular) layers | |  |
|  | Roundish cells: round to oval nucleated cells, at least twice the size of keratinocytes | | |
|  | Dendritic cells: bright nucleated cells with dendritic processes | | |
| Bright suprabasal epidermal areas | Areas at the spinous-granular layers of the epidermis showing hyper-reflective epidermal pattern, brighter than the surrounding normal epidermis | |  |
| DEJ pattern | Thick-Rings pattern: A low-magnification pattern of bright thick rim composed of single nucleated cells or as small junctional nests surrounding the dermal papillae | |  |
|  | Meshwork pattern: A low-magnification pattern composed of interconnecting bright, thickened and elongated tubular junctional structures | | |
|  | Clod pattern: A low-magnification pattern of large roundish aggregates of nests within the dermal-epidermal junction/superficial dermis | | |
| Interpapillary epidermis | Area of the basal and spinous epidermis, mostly displaying honeycomb or cobblestone pattern, which separates adjacent dermal papillae; this feature usually signifies epidermal rete ridges | |  |
|  | Normal: 2–4 layers of keratinocytes in between dermal papillae | | |
|  | Broadened: more than four layers of keratinocytes separating dermal papillae | | |
| Atypical cells | Presence of bright round or dendritic nucleated cells at DEJ that are abnormally large (more than twice the size of adjacent keratinocytes), and/or display an unusual contour | |  |
| Nest type | Dense homogeneous: compact aggregates with sharp margin and similar cells in morphology and refractivity | |  |
|  | Dense and sparse: aggregates with sharp and regular margins and homogeneity in cell morphology and refractivity, with the presence of evident cells not tightly aggregated within the nest | | |
|  | Heterogeneous/atypical: non-discrete aggregates of cells that differ in size, morphology and refractivity | | |
| Nest location | Junctional nests: Nests connected with the epidermal basal cell layer that may bulge into the dermal papillae | |  |
|  | Dermal nests: Nests located in dermal papilla/superficial dermis, without connection to the epidermal basal cell layer | | |
|  | Compound nests: Nests are seen in junctional as well as dermal distribution | | |
| DP-nest pattern | Dark: Dermal papillae are devoid of nests/aggregates | |  |
|  | Bulging: Junctional nests protrude into the dermal papillae with no to mild broadening of the dermal papillae | | |
|  | Expanded: Dermal papillae are markedly filled and expanded by junctional and/or dermal nests | | |
| DP-ring pattern | Edged (ringed): Dermal papillae surrounded by a rim of small bright cells | |  |
|  | Non-edged: Dermal papillae without a demarcated rim of bright cells | | |

**eTable 2. Description of demographic, clinical and dermoscopic data by diagnosis**

| CLINICAL PARAMETERS | | Total | SMs (n=33, 25.8%) | SN (n=95, 74.2%) | p-value |
| --- | --- | --- | --- | --- | --- |
|  |  | N (%) | N (%) | N (%) |  |
| Gender | Female | 83 (65%) | 21 (66%) | 62 (64%) | 0.795 |
|  | Male | 40 (31%) | 11 (34%) | 29 (30%) |  |
|  | Missing | 5 (4%) | 0 | 5 (5%) |  |
| Age | Mean ±SD (range) | 34.9 ±15.7(3-81) | 39.1 ±10.5(22-53) | 34.5 ±16.1(3-81) | 0.386 |
| Size (mm) | Mean ±SD (range) | 6.7 ±3.3(2-21) | 7.6 ±3.6(3-15) | 6.4 ±3.2(2-21) | 0.068 |
| Location | Head & Neck | 6 (5%) | 2 (6%) | 4 (4%) | 0.353 |
|  | Trunk | 26 (20%) | 5 (16%) | 21 (22%) |  |
|  | Upper extremity | 27 (21%) | 4 (12%) | 23 (24%) |  |
|  | Lower extremity | 69 (54%) | 21 (66%) | 48 (50%) |  |
| Colour | Skin colour | 2 (2%) | 0 | 2 (2%) | 0.149 |
|  | Pink to red | 17 (13%) | 5 (16%) | 12 (12.5%) |  |
|  | Light brown to tan | 31 (24%) | 12 (37.5%) | 19 (20%) |  |
|  | Medium brown to black | 78 (61%) | 15 (47%) | 63 (66%) |  |
| Palpability | Flat: macule or patch | 64 (50%) | 17 (53%) | 47 (49%) | 0.552 |
|  | Raised: papule or plaque | 49 (38%) | 10 (31%) | 39 (41%) |  |
|  | Nodular (nodule/tumor) | 15 (12%) | 5 (16%) | 10 (10%) |  |
| DERMOSCOPIC PARAMETERS | |  |  |  |  |
| Colour | Darkly pigmented | 91 (71%) | 20 (62.5%) | 71 (74%) | 0.425 |
|  | Lightly pigmented | 23 (18%) | 8 (25%) | 15 (16%) |  |
|  | Amelanotic | 14 (11%) | 4 (12.5%) | 10 (10%) |  |
| Blue-white veil | Absent | 62 (48.4%) | 18 (56.2%) | 44 (45.8%) | 0.589 |
|  | < 30% (focal) | 32 (25.0%) | 7 (21.8%) | 25 (26.0%) |  |
|  | ≥ 30% (diffuse) | 34 (26.6%) | 7 (21.8%) | 27 (28.1%) |  |
| NPN (Presence and extent) | Absent | 68 (53.1%) | 12 (37.5%) | 56 (58.3%) | **0.041** |
|  | < 30% (focal) | 12 (9.4%) | 2 (6.2%) | 10 (10.4%) |  |
|  | ≥ 30% (diffuse) | 48 (37.5%) | 18 (56.2%) | 30 (31.2%) |  |
| Shiny white structures | Present | 50 (39%) | 16 (50%) | 34 (35%) | 0.143 |
|  | Absent | 78 (61%) | 16 (50%) | 62 (64%) |  |
| Pattern type | Globular | 16 (12.5%) | 2 (6%) | 14 (15%) | 0.063 |
|  | Starburst | 8 (6%) | 1 (3%) | 7 (7%) |  |
|  | Reticular | 22 (17%) | 9 (28%) | 13 (13.5%) |  |
|  | Pigmented homogeneous | 35 (27%) | 4 (12.5%) | 31 (32%) |  |
|  | Amelanotic homogenous | 23 (18%) | 8 (25%) | 15 (16%) |  |
|  | Multicomponent | 24 (19%) | 8 (25%) | 16 (17%) |  |
| Pattern symmetry | Symmetric | 37 (29%) | 4 (12.5%) | 33 (34%) | **0.018** |
|  | Asymmetric | 91 (71%) | 28 (87.5%) | 63 (66%) |  |
| Blood vessels | No | 89 (69.5%) | 18 (56%) | 71 (74%) | 0.219 |
|  | Dotted | 27 (21%) | 10 (31%) | 17 (18%) |  |
|  | Linear | 0 (0%) | 0 (0%) | 0 (0%) |  |
|  | Polymorphous | 7 (5.5%) | 3 (9%) | 4 (4%) |  |
|  | Other | 5 (4%) | 1 (3%) | 4 (4%) |  |
|  |  |  |  |  |  |

Abbreviations: SM, Spitzoid melanoma; SN, Spitz naevus; SD, standard deviation; OR, odds ratio; CI, confidence interval.

**eTable 3. Description of RCM data by diagnosis**

| RCM PARAMETERS | | Total | SMs (n=33, 25.8%) | SN (n=95, 74.2%) | p-value |
| --- | --- | --- | --- | --- | --- |
| Linear surface grooves | Yes | 55 (43%) | 16 (50%) | 39 (41%) | 0.256 |
|  | No | 69 (54%) | 14 (44%) | 55 (57%) |  |
|  | Missing | 4 (3%) | 2 (6%) | 2 (2%) |  |
| Round surface holes | Yes | 92 (72%) | 22 (69%) | 70 (73%) | 0.902 |
|  | No | 32 (25%) | 8 (25%) | 24 (25%) |  |
|  | Missing | 4 (3%) | 2 (6%) | 2 (2%) |  |
| Predominant epidermis pattern | Typical honeycomb/cobblestone pattern | 61 (47.5%) | 15 (47.5%) | 46 (48%) | 0.407 |
|  | Atypical honeycomb/cobblestone pattern | 37 (41%) | 11 (27%) | 41 (43%) |  |
|  | Disarranged | 13 (10%) | 6 (19%) | 7 (7%) |  |
|  | Missing | 2 (2%) | 0 (0%) | 2 (2%) |  |
| Bright suprabasal epidermal areas | Absent | 53 (41%) | 15 (47%) | 38 (40%) | 0.529 |
|  | Present | 74 (16%) | 17 (19%) | 57 (16%) |  |
|  | Missing | 1 (1%) | 0 (0%) | 1 (1%) |  |
| Roundish cells in suprabasal epidermis | Absent | 103 (82.4%) | 24 (77.4%) | 79 (84.0%) | 0.088 |
|  | < 30% | 19 (15.2%) | 5 (16.1%) | 14 (14.9%) |  |
|  | ≥ 30% | 3 (2.4%) | 2 (6.4%) | 1 (1.0%) |  |
| Dendritic cells in suprabasal epidermis | Absent | 63 (49.2%) | 15 (46.9%) | 48 (50.0%) | 0.495 |
|  | < 30% | 42 (32.8%) | 9 (28.1%) | 33 (34%) |  |
|  | ≥ 30% | 19 (15.6%) | 7 (21.9%) | 13 (13.5%) |  |
|  | Missing | 3 (2.3%) | 1 (3.1%) | 2 (2.1%) |  |
| Predominant DEJ pattern | Thin ring | 11 (9%) | 4 (12.5%) | 7 (7%) | 0.178 |
|  | Thick ring | 43 (34%) | 14 (44%) | 29 (30%) |  |
|  | Meshwork | 29 (23%) | 3 (9%) | 26 (27%) |  |
|  | Clods | 28 (22%) | 5 (16%) | 23 (24%) |  |
|  | Nonspecific/disarranged | 11 (9%) | 3 (9%) | 8 (8%) |  |
|  | Missing | 6 (5%) | 3 (9%) | 3 (3%) |  |
| Thin rings | < 30% | 104 (81%) | 23 (72%) | 81 (84%) | 0.302 |
|  | ≥ 30% | 18 (14%) | 6 (19%) | 12 (12.5%) |  |
| Thick rings | < 30% | 67 (52%) | 13 (41%) | 54 (56%) | 0.211 |
|  | ≥ 30% | 55 (43%) | 16 (50%) | 39 (41%) |  |
| Meshwork | < 30% | 80 (62%) | 24 (75%) | 56 (58%) | **0.026** |
|  | ≥ 30% | 42 (33%) | 5 (16%) | 37 (38.5%) |  |
| Clods | < 30% | 87 (68%) | 23 (72%) | 64 (67%) | 0.275 |
|  | ≥ 30% | 35 (27%) | 6 (19%) | 29 (30%) |  |
| Non-specific | < 30% | 76 (59%) | 15 (47%) | 61 (63.5%) | 0.179 |
|  | ≥ 30% | 46 (36%) | 14 (44%) | 32 (33%) |  |
| Interpapillary epidermis | Regular | 18 (14%) | 5 (16%) | 13 (13.5%) | 0.678 |
|  | Broadened | 99 (77%) | 23 (72%) | 76 (79%) |  |
|  | Missing | 11 (9%) | 4 (12.5%) | 7 (7%) |  |
| Atypical cells at DEJ | Absent | 74 (57.8%) | 17 (53.1%) | 57 (59.4%) | 0.776 |
|  | < 30% | 30 (23.4%) | 7 (21.8%) | 23 (23.9%) |  |
|  | ≥ 30% | 16 (12.5%) | 5 (15.6%) | 11 (11.5%) |  |
|  | Missing | 8 (6.2%) | 3 (9.4%) | 5 (5.2%) |  |
| Nests - predominant type | Dense homogeneous | 42 (33%) | 9 (28%) | 33 (34%) | 0.174 |
|  | Dense and sparse | 13 (10%) | 1 (3%) | 12 (12.5%) |  |
|  | Heterogeneous/atypical | 38 (29%) | 12 (37.5%) | 26 (27%) |  |
|  | Missing | 35 (27%) | 10 (29%) | 25 (26%) |  |
| Nests – predominant location | Junctional | 72 (56%) | 15 (47%) | 57 (59%) | 0.391 |
|  | Dermal | 11 (9%) | 4 (12.5%) | 7 (7%) |  |
|  | Compound | 12 (9%) | 4 (12.5%) | 8 (8%) |  |
|  | Missing | 33 (26%) | 9 (28%) | 24 (25%) |  |
| DP-nests predominant pattern | Dark | 52 (41%) | 17 (53%) | 35 (36.5%) | **0.022** |
|  | Bulging | 38 (30%) | 3 (9%) | 35 (36.5%) |  |
|  | Expanded | 12 (9%) | 5 (16%) | 7 (7%) |  |
|  | Nonspecific | 14 (11%) | 3 (9%) | 11 (11.5%) |  |
|  | Missing | 12 (9%) | 4 (12.5%) | 8 (8%) |  |
| Dark | < 30% | 37 (29%) | 7 (22%) | 30 (31%) | 0.366 |
|  | ≥ 30% | 71 (55.5%) | 19 (59%) | 52 (54%) |  |
| Bulging | < 30% | 54 (42%) | 18 (56%) | 36 (37.5%) | **0.019** |
|  | ≥ 30% | 56 (44%) | 8 (25%) | 48 (50%) |  |
| Expanded | <30 | 89 (69.5%) | 21 (65.6%) | 68 (71%) | 0.983 |
|  | ≥30 | 21 (16%) | 5 (16%) | 16 (17%) |  |
| Non-specific | < 30% | 68 (53%) | 15 (47%) | 53 (55%) | 0.571 |
|  | ≥ 30% | 41 (32%) | 11 (34%) | 30 (31%) |  |
| DP-ring pattern | Edged | 33 (26%) | 11 (34%) | 22 (23%) | 0.107 |
|  | Non-edged | 61 (48%) | 10 (31%) | 51 (53%) |  |
|  | Mixed | 21 (16%) | 7 (22%) | 14 (15%) |  |
|  | Missing | 13 (10%) | 4 (12.5%) | 9 (9%) |  |

Abbreviations: SM, Spitzoid melanoma; SN, Spitz naevus; SD, standard deviation; OR, odds ratio; CI, confidence interval.

**eTable 4. Univariate and multivariate models for diagnosis by clinical, dermoscopic and RCM parameters**

|  |  | Univariate analysis | | Multivariate analysis | | |
| --- | --- | --- | --- | --- | --- | --- |
| CLINICAL PARAMETERS |  | OR 95%CI | p-value | OR 95%CI | p-value |  |
| Size | <5 mm | ref. |  |  |  |  |
|  | ≥ 5 mm, <10mm | 1.83 (0.65-5.12) | 0.248 |  |  |  |
|  | ≥ 10 mm | 4.12 (1.21-14.05) | **0.024** |  |  |  |
| Location | head & neck | ref. |  |  |  |  |
|  | trunk | 0.47 (0.06-3.37) | 0.458 |  |  |  |
|  | upper extremity | 0.34 (0.04-2.57) | 0.301 |  |  |  |
|  | lower extremity | 0.87 (0.14-5.15) | 0.883 |  |  |  |
| Colour | skin colour | ref. |  |  |  |  |
|  | pink to red | - | - |  |  |  |
|  | light brown to tan | 2.65 (1.06-6.63) | **0.037** |  |  |  |
|  | medium brown to black | 1 | - |  |  |  |
| Palpability | flat | ref. |  |  |  |  |
|  | palpable | 0.70 (0.29-1.72) | 0.448 |  |  |  |
|  | nodular | 1.38 (0.41-4.62) | 0.599 |  |  |  |
| Palpability | No | ref. |  |  |  |  |
|  | Yes | 1.02 (0.57-1.82) | 0.941 |  |  |  |
| DERMOSCOPIC PARAMETERS |  |  |  |  |  |  |
| NPN | absent | ref. |  |  |  |  |
|  | present | 2.33 (1.02-5.31) | **0.044** |  |  |  |
| Colour | darkly pigmented | ref. |  |  |  |  |
|  | lightly-pigmented | 1.89 (0.70-5.10) | 0.207 |  |  |  |
|  | amelanotic | 1.42 (0.40-5.01) | 0.586 |  |  |  |
| Blue-white structures | absent | ref. |  | ref. |  |  |
|  | < 30% | 0.69 (0.25-1.86) | 0.458 | 0.25 (0.07-0.86) | **0.027** |  |
|  | ≥ 30% | 0.63 (0.23-1.71) | 0.369 | - | - |  |
| Shiny white structures | absent | ref. |  |  |  |  |
|  | present | 1.82 (0.81-4.09) | 0.146 |  |  |  |
| Pattern type | multicomponent | ref. |  |  |  |  |
|  | globular | 0.28 (0.05-1.57) | 0.150 |  |  |  |
|  | starburst | 0.28 (0.02-2-73) | 0.277 |  |  |  |
|  | reticular | 1.38 (0.41-4.60) | 0.595 |  |  |  |
|  | pigmented homogeneous | 0.25 (0.06-0.98) | **0.048** |  |  |  |
|  | amelanotic homogenous | 1.06 (0.31-3.56) | 0.917 |  |  |  |
| Pattern symmetry | symmetric | ref. |  |  |  |  |
|  | asymmetric | 3.66 (1.18-11.34) | **0.024** |  |  |  |
| Blood vessels | no blood vessels | ref. |  |  |  |  |
|  | dotted | 2.32 (0.90-5.92) | 0.078 |  |  |  |
|  | linear | - | - |  |  |  |
|  | polymorphous | 2.95 (0.60-14.41) | 0.180 |  |  |  |
|  | other | 0.98 (0.10-9.37) | 0.990 |  |  |  |
| RCM PARAMETERS |  |  |  |  |  |  |
| Linear surface grooves | absent | ref. |  |  |  |  |
|  | present | 0.61 (0.70-3.68) | 0.258 |  |  |  |
| Round surface holes | Absent | ref. |  |  |  |  |
|  | Present | 0.94 (0.37-2.39) | 0.902 |  |  |  |
| Predominant Epidermis pattern | Typical | ref. |  |  |  |  |
|  | Atypical | 0.60 (0.24-1.52) | 0.291 |  |  |  |
|  | Disarranged | 2.05 (0.55-7.67) | 0.283 |  |  |  |
| Bright suprabasal epidermal areas | Absent | ref. |  |  |  |  |
|  | Present | 0.75 (0.33-1.69) | 0.496 |  |  |  |
| Roundish cells in suprabasal layer | absent | ref. |  |  |  |  |
|  | < 30% | 1.17 (0.38-3.59) | 0.777 |  |  |  |
|  | ≥ 30% | 6.58 (0.57-75.79) | 0.131 |  |  |  |
| Dendritic cells in suprabasal layer | absent | ref. |  |  |  |  |
|  | < 30% | 0.87 (0.34-2.22) | 0.776 |  |  |  |
|  | ≥ 30% | 1.72 (0.58-5.10) | 0.326 |  |  |  |
| Predominant DEJ pattern | thin ring | ref. |  |  |  |  |
|  | thick ring | 0.84 (0.21-3.37) | 0.811 |  |  |  |
|  | meshwork | 0.20 (0.03-1.12) | 0.067 |  |  |  |
|  | clods | 0.38 (0.07-1.81) | 0.226 |  |  |  |
|  | nonspecific/disarranged | 0.65 (0.10-4.00) | 0.648 |  |  |  |
| Interpapillary epidermis | regular | ref. |  |  |  |  |
|  | broadened | 0.78 (0.25-2.44) | 0.678 |  |  |  |
| Atypical cells at DEJ | absent | ref. |  | ref. |  |  |
|  | < 30% | 1.02 (0.37-2.78) | 0.968 | 3.29 (1.14-9.43) | **0.027** |  |
|  | ≥ 30% | 1.52 (0.46-4.99) | 0.487 | - | - |  |
| Nests - predominant type | dense homogeneous | ref. |  |  |  |  |
|  | dense and sparse | 0.33 (0.03-2.93) | 0.322 |  |  |  |
|  | heterogeneous/atypical | 1.69 (0.61-4.62) | 0.305 |  |  |  |
| Nests - predominant location | junctional | ref. |  |  |  |  |
|  | dermal | 2.17 (0.56-8.40) | 0.262 |  |  |  |
|  | compound | 1.9 (0.50-7.17) | 0.344 |  |  |  |
| DP-nests predominant pattern | dark | ref. |  |  |  |  |
|  | bulging | 0.17 (0.04-0.64) | 0.10 |  |  |  |
|  | expanded | 1.47 (0.40-5.31) | 0.557 |  |  |  |
|  | nonspecific | 0.56 (0.14-2.28) | 0.420 |  |  |  |
| DP-ring pattern | edged | ref. |  |  |  |  |
|  | non-edged | 0.39 (0.14-1.05) | 0.064 |  |  |  |
|  | mixed | 1 (0.31-3.19) | 1.000 |  |  |  |

Abbreviations: RCM, reflectance confocal microscopy; OR, odds ratio; CI, confidence interval; DEJ, dermal-epidermal junction; DP, dermal papillae.

**eTable 5. Frequency of histopathological criteria by diagnosis**

| HISTOPAHOLOGICAL CRITERIA | No (0) | SN | SMs | Total |
| --- | --- | --- | --- | --- |
|  | Yes (1) |  |  |  |
| Orthokeratosis | 0 | 2 (15%) | 3 (43%) | 5 (25%) |
|  | 1 | 11 (85%) | 4 (57%) | 15 (75%) |
| Keratin-filled surface dells | 0 | 2 (15%) | 2 (29%) | 4 (20%) |
|  | 1 | 11 (85%) | 5 (71%) | 16 (80%) |
| Hypergranulosis | 0 | 7 (54%) | 2 (29%) | 9 (45%) |
|  | 1 | 6 (46%) | 5 (71%) | 11 (55%) |
| Wedge-shaped hypergranulosis | 0 | 8 (62%) | 3 (43%) | 11 (55%) |
|  | 1 | 5 (38%) | 4 (57%) | 9 (45%) |
| Acanthosis | 0 | 2 (15%) | 1 (14%) | 3 (15%) |
|  | 1 | 11 (85%) | 6 (86%) | 17 (85%) |
| Irregular epidermal hyperplasia | 0 | 3 (23%) | 1 (14%) | 4 (20%) |
|  | 1 | 10 (77%) | 6 (86%) | 16 (80%) |
| Broadened infundibula | 0 | 3 (23%) | 0 (0%) | 3 (15%) |
|  | 1 | 10 (77%) | 7 (100%) | 17 (85%) |
| Broadened retes | 0 | 3 (23%) | 0 (0%) | 3 (15%) |
|  | 1 | 10 (77%) | 7 (100%) | 17 (85%) |
| Dermal fibroplasia | 0 | 4 (31%) | 3 (43%) | 7 (35%) |
|  | 1 | 9 (69%) | 4 (57%) | 13 (65%) |

Abbreviations: SM, Spitzoid melanoma; SN, Spitz naevus

*
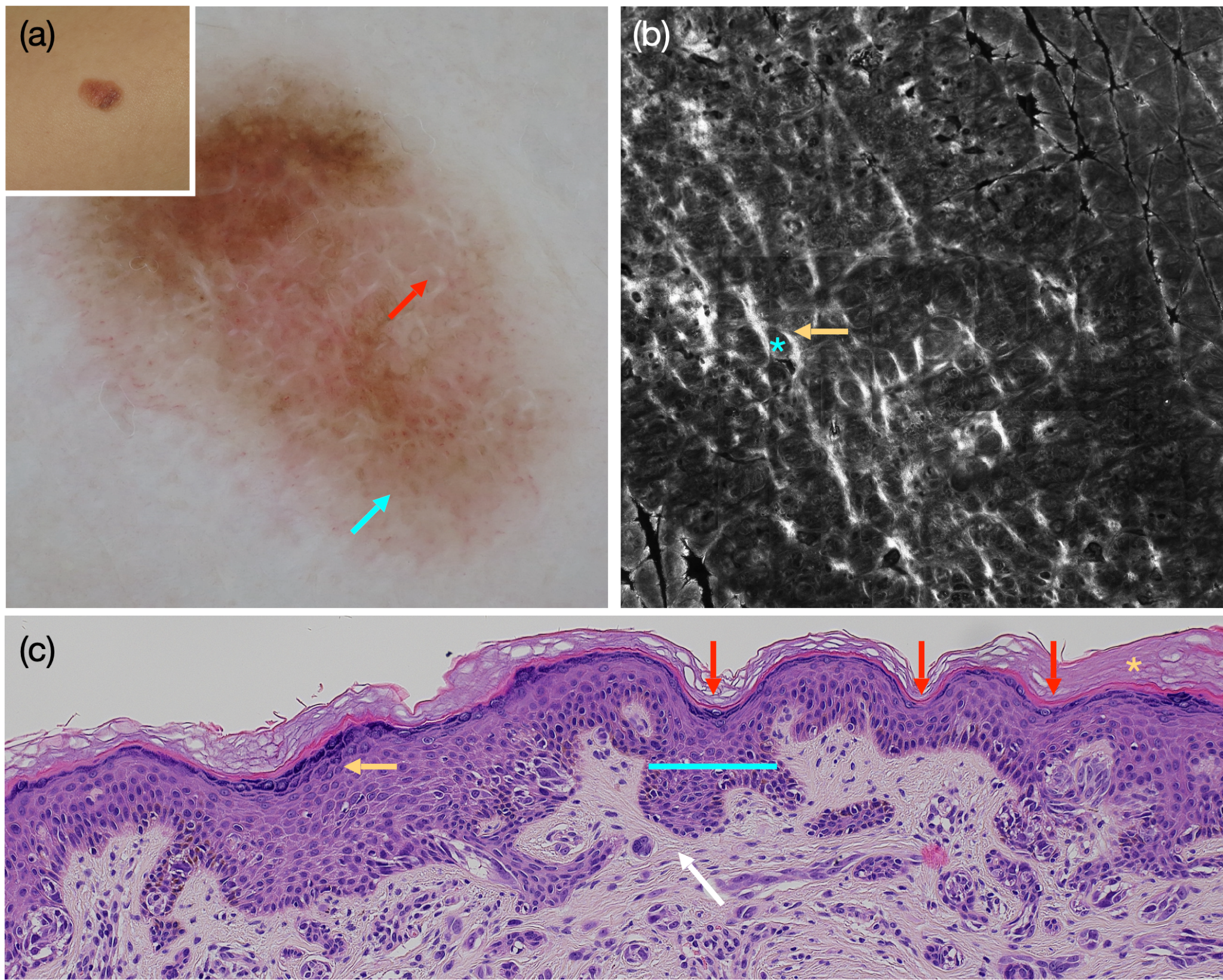
*

**eFigure 1. Spitz naevus**, on the arm of a 36-year-old woman. (a) Inset showing an asymmetric light brown papule. Dermoscopy showing multicomponent pattern with a negative pigment network (blue arrow) and shiny white lines (red arrow), as well as asymmetry in the distribution of colours. (b) RCM at the DEJ level shows dark dermal papillae (blue asterisk) separated by strand-like bright suprabasal epidermal areas (yellow arrow). Note that the polarization of the epidermis echoes that of the shiny white lines seen on dermoscopy. (c) Histopathology shows hyperkeratosis (yellow asterisk), wavy surface of the epidermis with multiple keratin-filled surface dells (red arrows), hypergranulosis (yellow arrow), and broadening of the interpapillary retes (blue line). The superficial dermis shows fibrosis (white arrow).

*
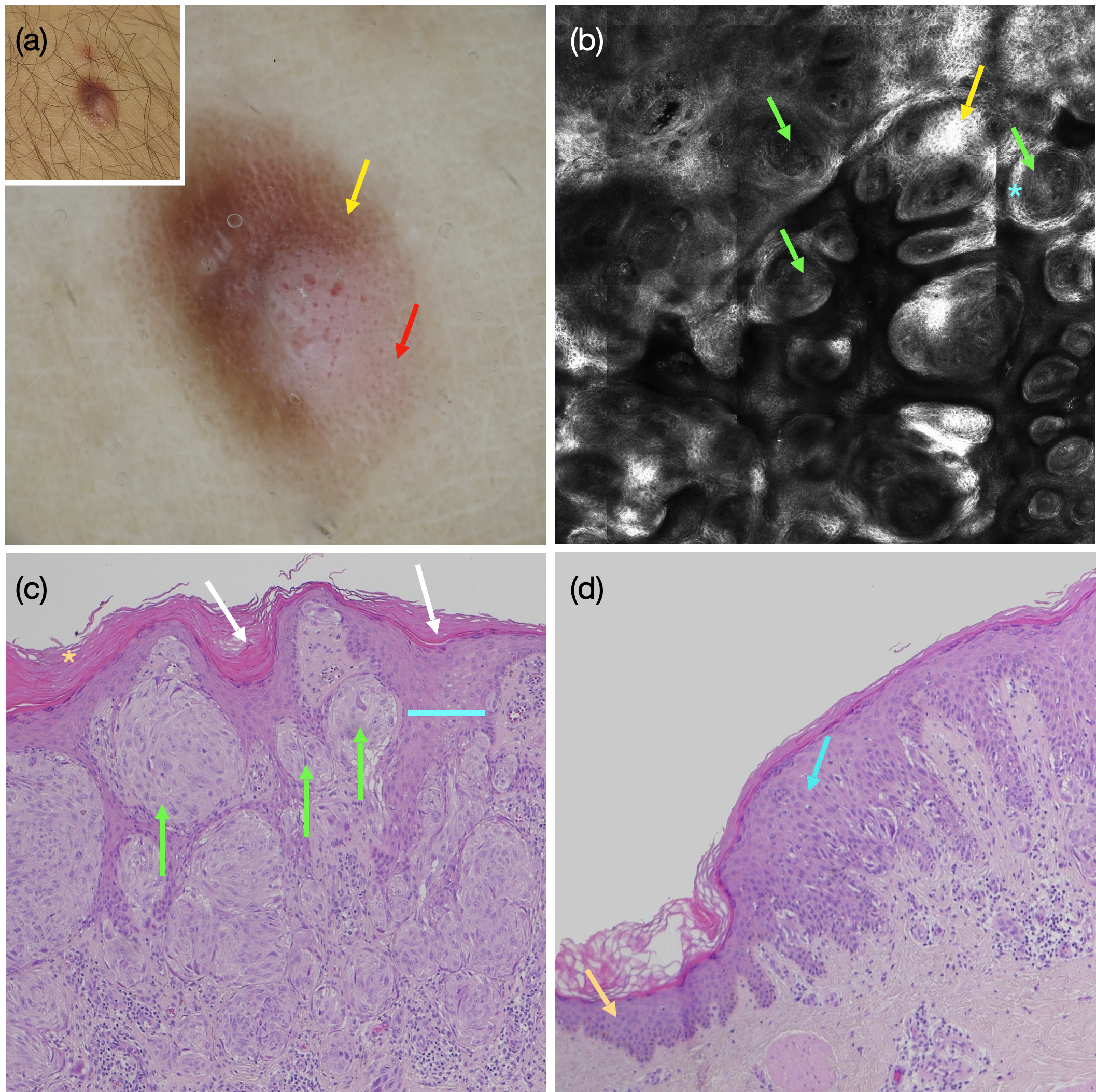
*

**eFigure 2. Spitzoid melanoma 1.2 mm in thickness**, on the leg of a 22-year-old man. (a) Inset showing an asymmetric contour composed of a non-pigmented raised papule and a light-brown flat papule. Dermoscopy shows an asymmetric multicomponent pattern with negative pigment network (yellow arrow) and dotted vessels (red arrow). (b) RCM at the spinous-granular layers shows bright suprabasal epidermal areas (yellow arrow) and slightly refractile nests (green arrows) that appear “cuffed” by the bright surrounding epidermis. (c) Histopathology shows hyperkeratosis (yellow asterisk) and keratin-filled surface dells (white arrows), broadened and elongated retes (blue line) closely "cuffing" the junctional nests that protrude into the superficial epidermis (green arrows). (d) Note the hyperplasia of keratinocytes in the lesion (blue arrow), compared to the surrounding skin (yellow arrow).

*
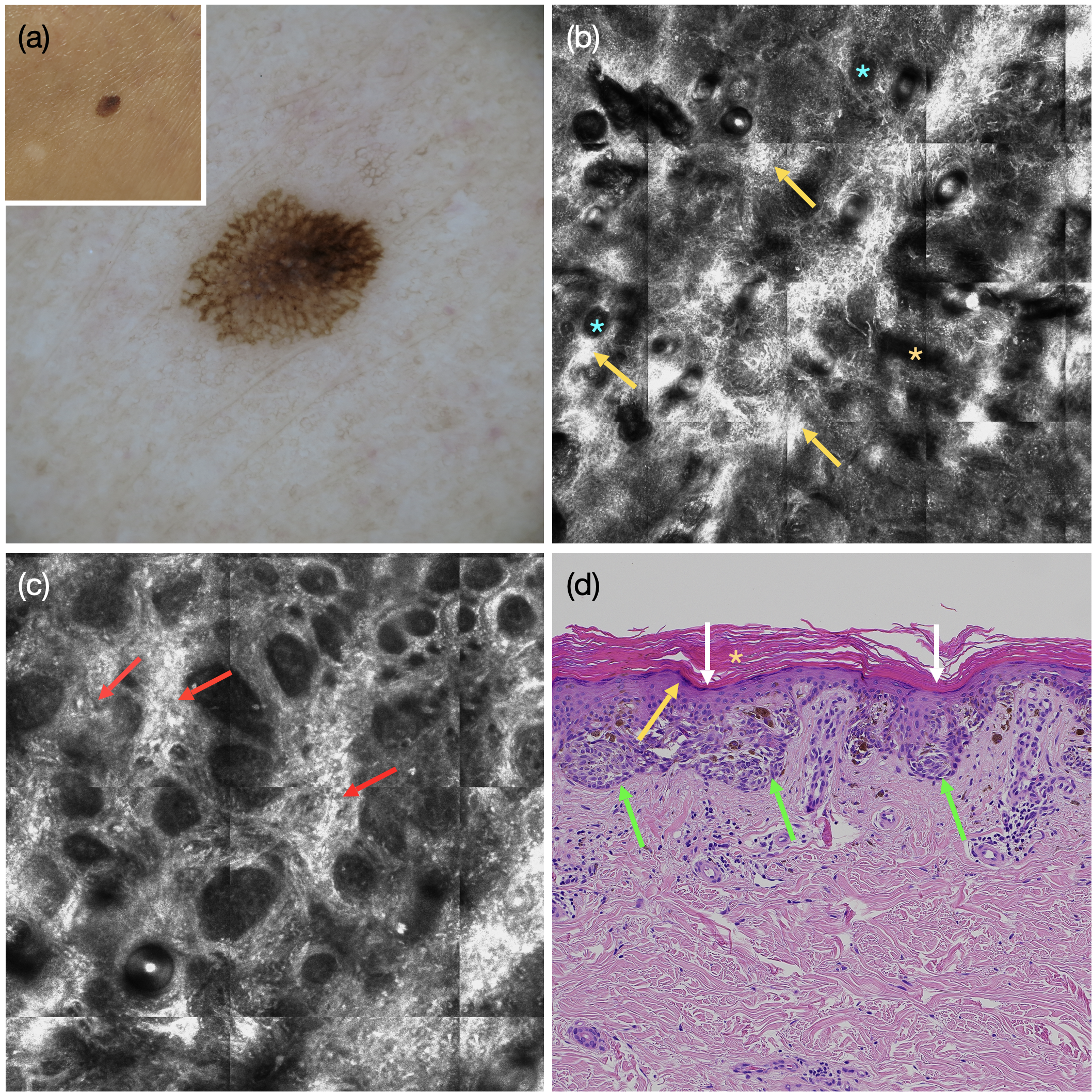
*

**eFigure 3. Spitzoid melanoma 0.2 mm in thickness**, on the leg of a 68-year-old man. (a) Inset showing a brown macule. Dermoscopy showing irregular network with peripheral streaks; a negative pigment network is not seen. (b) RCM at the spinous-granular layers shows round surface holes (blue asterisks), linear surface grooves (yellow asterisk), and bright suprabasal epidermal areas (yellow arrows). (c) RCM at the DEJ level shows a meshwork pattern, irregular in thickeness, composed of non-homogeneous aggregates of bright dendritic melanocytes (arrows) (d) Corresponding histopathology shows undulating surface with hyperkeratosis (yellow asterisk) with keratin-filled surface dells (white arrows), hypergranulosis (yellow arrow), and junctional discohesive nests of melanocytes that are rimmed by basal keratinocytes (green arrows).
